# Mathematical Model of a Cell Size Checkpoint

**DOI:** 10.1371/journal.pcbi.1001036

**Published:** 2010-12-16

**Authors:** Marco Vilela, Jeffrey J. Morgan, Paul A. Lindahl

**Affiliations:** 1 Department of Chemistry, Texas A&M University, College Station, Texas, United States of America; 2 Department of Mathematics, University of Houston, Houston, Texas, United States of America; 3 Department of Biochemistry and Biophysics, Texas A&M University, College Station, Texas, United States of America; University of Virginia, United States of America

## Abstract

How cells regulate their size from one generation to the next has remained an enigma for decades. Recently, a molecular mechanism that links cell size and cell cycle was proposed in fission yeast. This mechanism involves changes in the spatial cellular distribution of two proteins, Pom1 and Cdr2, as the cell grows. Pom1 inhibits Cdr2 while Cdr2 promotes the G2 → M transition. Cdr2 is localized in the middle cell region (midcell) whereas the concentration of Pom1 is highest at the cell tips and declines towards the midcell. In short cells, Pom1 efficiently inhibits Cdr2. However, as cells grow, the Pom1 concentration at midcell decreases such that Cdr2 becomes activated at some critical size. In this study, the chemistry of Pom1 and Cdr2 was modeled using a deterministic reaction-diffusion-convection system interacting with a deterministic model describing microtubule dynamics. Simulations mimicked experimental data from wild-type (WT) fission yeast growing at normal and reduced rates; they also mimicked the behavior of a Pom1 overexpression mutant and WT yeast exposed to a microtubule depolymerizing drug. A mechanism linking cell size and cell cycle, involving the downstream action of Cdr2 on Wee1 phosphorylation, is proposed.

## Author Summary

Cells delay division into two daughter cells until they reach a particular size. However, the molecular-level mechanisms by which they do this have remained unknown until recently. A cell-size checkpoint mechanism in rod-shaped fission yeast cells has recently been shown to involve two proteins, Pom1 and Cdr2. The concentrations of these proteins in the middle of the cell differ from that at the poles. The changing nature of these spatial gradients as the cell grows is size-sensitive. Pom1 inhibits Cdr2 while Cdr2 stimulates the cell to enter into mitosis. In short cells, the Pom1 concentration in the middle of the cell is so great that Cdr2 is inhibited. As cells grow, the Pom1 concentration in the middle of the cell declines; at some particular size, Cdr2 activates. In this study, we developed a mathematical model that mimics this checkpoint behavior.

## Introduction

Dividing cells maintain a stable size from one generation to the next. This suggests that they contain homeostatic mechanisms in which the division cycle is triggered when a particular size is attained. However, the biochemical mechanisms for this have remained unknown puzzles for decades. Sensing mechanisms appear restricted to monitoring *concentration* changes, so how can such changes reflect cell volume? Volume and concentration are different *types* of quantities; the former is sensitive to changes in scale while the latter is not. This issue has been discussed [1] and possibilities have been proposed. Most of these involve measuring the time required for a cellular component to reach a critical concentration beyond which mitosis is triggered [1], [2].

**Figure 1:**
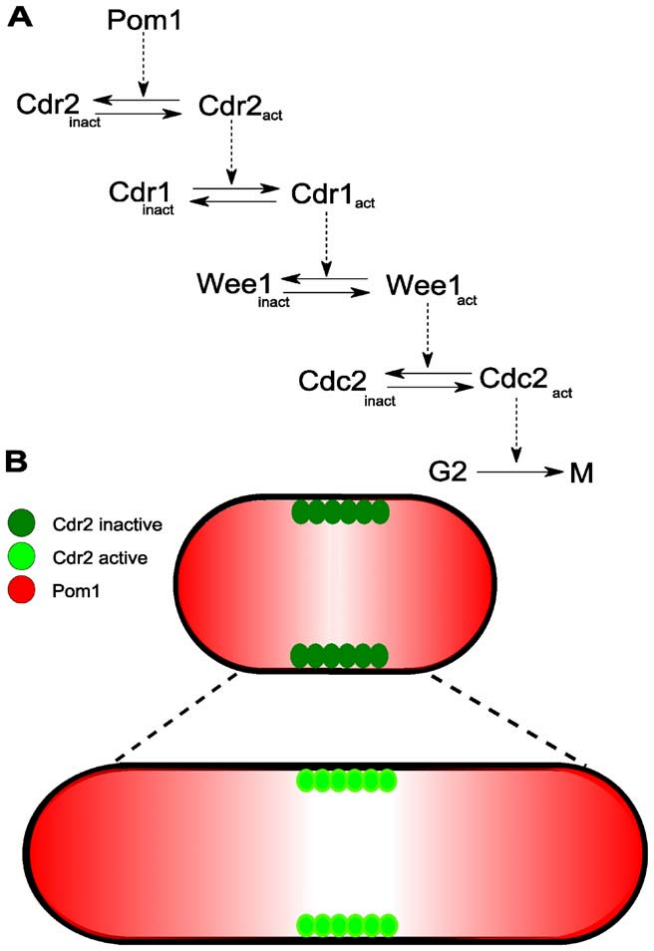
Mechanism of a cell-size checkpoint involving Pom1 and Cdr2. (A) Reaction network leading to the activation of mitosis in fission yeast. (B) Observed spatial distributions of Pom1 and Cdr2 in short and long cells [4], [5]. Pom1 concentration (red) is highest at the poles and lowest in the midcell region where Cdr2 concentrates in cortical nodes (green). In early interphase (short cells), Pom1 at the midcell is present at a sufficient concentration to inhibit Cdr2 and the G2/M transition *via* the cascade in (A). As cells grow, the midcell Pom1 concentration decreases until it crosses a threshold that relieves Cdr2 inhibition thereby promoting the G2/M transition.

Because their cell-length phenotypes are directly linked to the time spent in specific cell cycle stages, fission yeast *Schizosaccharomyces pombe* are especially useful in understanding the relationship between cell size and cell cycle [3]. These 7 µm long rod-shaped newborn cells grow lengthwise to ∼14 µm at which point they divide. A mechanistic model of how these cells might sense size, involving Pom1 and Cdr2 proteins as major players, was recently proposed [4], [5]. Pom1 is a kinase involved in cell polarization and in establishing the cell division plane [6], [7], [8]. Cdr2 is a serine-threonine protein kinase that promotes the G2/M transition by inactivating Wee1, an inhibitor of Cdc2 [3], [9], [10]. In the proposed mechanism, Pom1 inhibits Cdr2. The size-dependent relief of this inhibition indirectly activates Cdc2, which promotes entry into mitosis (Figure 1A) [4], [5].

The cell-size-dependence of Pom1 and Cdr2 are proposed to originate from the *relative spatial distributions of the two proteins*. Pom1 forms a spatial gradient that peaks at the cell tips and decreases towards the middle of the cell (midcell) (Figure 1B). This gradient arises from an indirect interaction with microtubules (MTs), mediated through the Tea1 protein [6], [7], [11]. During interphase, Tea1 is transported from the nuclear region of the cell to the tips by both “walking” along microtubules and by “riding” on microtubules' growing ends [11], [12], [13]. Microtubules occasionally undergo catastrophic collapse, releasing Tea1 in the process. Catastrophe occurs with higher frequency at the tips, causing Tea1 to be delivered preferentially to these regions [14], [15]. Tea1 anchors to the membrane in a complex positive-feedback process [16], [17]. Anchored Tea1 recruits Pom1 from the cytosol, sequestering it to the membrane and giving rise to the Pom1 spatial gradient. Conversely, Cdr2 is found in cortical node-like structures on the cell membrane in the midcell region during interphase. Midcell localization appears to be Pom1-dependent, because in cells lacking Pom1, Cdr2 spreads broadly from midcell to the non-growing end [4], [5].

The Pom1 gradient is present throughout interphase, but the concentration of Pom1 at midcell is length- (and thus size-) sensitive. During early interphase, the Pom1 concentration at midcell is sufficiently high to inhibit Cdr2 from advancing the cell from G2- to M-phase. When the cell reaches a particular length, the Pom1 midcell concentration declines enough for this inhibition to be relieved. This allows Cdr2 to trigger a cascade (Figure 1A) that ultimately advances the cell to the M-phase of mitosis.

Previous mathematical models have described the control of the G2/M transition [18], [19], [20], [21]. Although some describe the main cell cycle proteins in detail, none includes a specific mechanism for measuring cell size. Here we propose a simple 1D reaction-diffusion-convection mathematical model for a cell-size checkpoint based on the recently proposed Pom1:Cdr2 mechanism. We have attempted to make the model minimal in terms of the assumptions, reactions and components required to exhibit checkpoint behavior as arising from the spatial cellular dynamics of Pom1 and Cdr2 during interphase. The framework combines known chemical features of Pom1 and Cdr2 with the known dynamics of microtubules. The model reproduces phenotypes of a mutant fission yeast strain as well as the effects of two drugs. Our simulations demonstrate that the proposed checkpoint mechanism is feasible from a quantitative perspective.

## Model

The model was designed as three interacting subsystems, including a) Pom1:Cdr2 spatial gradients, b) the microtubule subsystem, and c) the triggering of mitosis. Details of each subsystem and how they interact within the context of a growing cell are included below, and reactions are given in Table 1. In general, the desired growth rate α was used to calculate how the volume (and thus length) of a cylindrically shaped cell changes with time. This information was used by the microtubule subsystem to generate a time-dependent distribution of microtubules of all different lengths. This distribution was inputted into the Pom1:Cdr2 subsystem, affording length-dependent spatial gradients for Pom1. These gradients, in turn, were used to obtain the associated Cdr2 spatial gradients. The averaged concentration of Cdr2 in the midcell region was used as a trigger for mitosis.

**Table 1:** Details of the chemical model.

Name	Reaction	Rate Expression	Kinetic Parameters
	**Pom1:Cdr2 Subsystem**		
Pom1 production	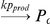	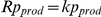	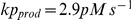
Pom1 partitioning	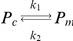	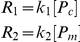	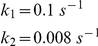
Cdr2 production	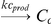	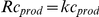	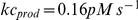
Cdr2 membrane insertion	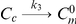	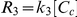	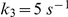
Cdr2 phosphorylation	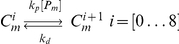	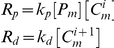	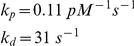
Cdr2 membrane expulsion	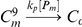	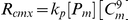	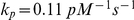
	**Microtubule Subsystem**		
Tubulin production	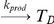	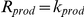	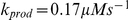
Tubulin Nucleotide exchange	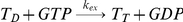	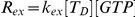	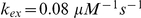
Tubulin Nucleation	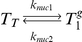	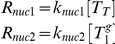	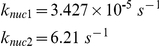
Microtubule Elongation	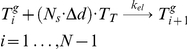	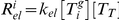	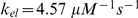
Microtubule Catastrophe	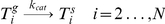	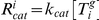	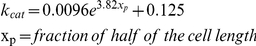
Microtubule Depolymerization	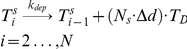	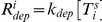	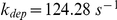
	**Trigger Subsystem**		
Cdr2 trigger	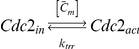	Goldbeter-Koshland equation 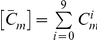	See [34]

### Pom1:Cdr2 Subsystem

**Figure 2:**
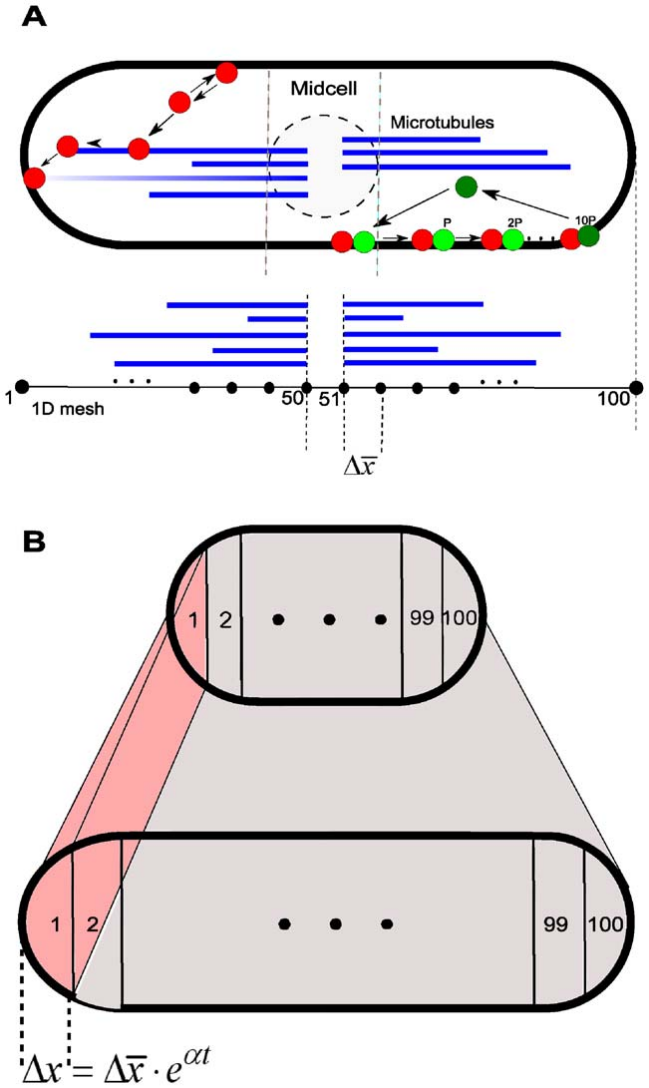
Modeling assumptions. (A) Pom1 moves toward the poles along microtubules; it attaches to and detaches from the membrane in a first-order process. Cdr2 attaches to the membrane by a first-order reaction but detaches only after being multiphosphorylated. The cell was discretized in a 1D mesh where the reaction-diffusion-convection system was solved. (B) Cells of all lengths were divided into 100 mesh points along the cell poles. Each mesh point represents a region of the cell that increases exponentially with time.

The Pom1:Cdr2 reaction-diffusion-convection model assumes diffusion along a 1D mesh (Figure 2A). This subsystem involves Pom1 and Cdr2 in cytosolic and membrane-bound forms (P_c_, P_m_, C_c_, C_m_). Membrane diffusion is significantly slower than cytosolic diffusion. Pom1 can partition between cytosolic and membrane-bound forms through an uncatalyzed reversible reaction. C_c_ inserts into the membrane where it is multiphosphorylated by Pom1. Once fully phosphorylated, Cdr2 is expelled from the membrane and simultaneously dephosphorylated. This mechanism assumes an ordered distributive chain of enzymatic reactions [22]. For simplicity, dephosphorylations are catalyzed by an unspecified and implicit phosphatase whose concentration is assumed to be constant throughout the cell cycle.

To model cell growth, equations were derived within a growing domain framework [23], [24]. Here, we fixed the 1D mesh length by normalizing the *x*-axis coordinate to cell length (L(t))
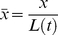
(1)Thus, one can ensure that a system described in this new coordinate 
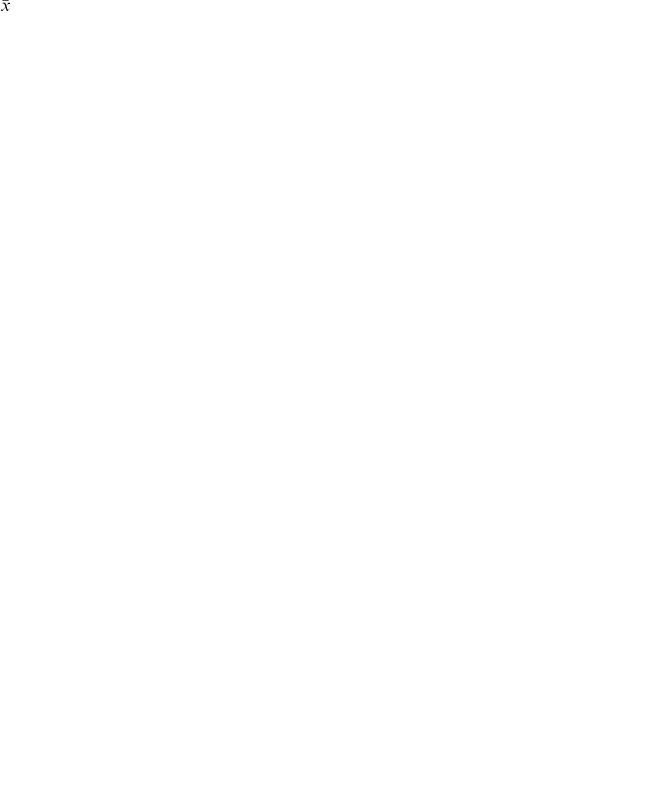
, is bounded within the interval 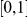 given that its domain is expressed in the old coordinate *x* by the function *L(t)*. In this fixed domain, the number of mesh points does not change with time such that standard numerical methods can be applied [23]. The interpretation is that the real cellular region represented by a given discretization point is growing. However, because of this fixed domain, the discretized interval 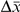 and the number of intervals used in the numerical calculations, namely 100, are invariant with time. This method reduces resolution but not precision (Figure 2B).

Given this fixed domain strategy and corresponding rates assigned for the reactions of the chemical model, the system can be described as
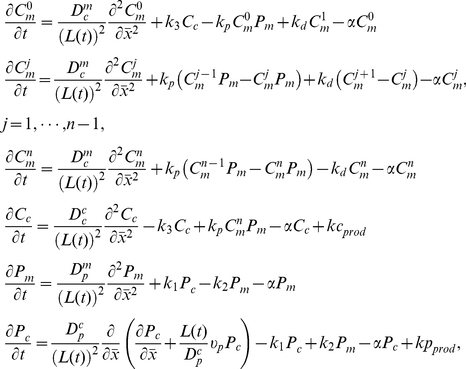
(2)Where
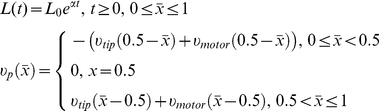
(3)subject to no-flux boundary conditions for all components and non-negative initial data. Subscript *n* refers to the total number of phosphorylation sites on Cdr2 and *j* refers to those that are phosphorylated. The denominator for each diffusion and convection term of system (2) arises from the spatial normalization of the growing domain. We assume exponential uniform growth at a time-invariant rate α (3). Because of the unidimensional mode of growth in fission yeast, only cell length *L(t)* was required to be modeled. Interfacial reactions (between cytosol and membrane) were normalized by the ratio of the surface area to cytosol volume. But because cell volume was approximated by a cylinder, this ratio remained constant, with growth exclusively along the long axis. This allowed us to embed this interfacial normalization ratio into reaction rate constants. The spatial system was numerically solved using the Crank-Nicolson method implemented in Fortran.

### Microtubule Subsystem

This subsystem involves microtubule reaction dynamics, which are required for generating the Pom1 and Crd2 spatial gradients. All assumed reactions are given in Table 1. Although microtubules actually consist of two tubulin isoforms (α and β), only one “lumped” isoform (T) was used in the model. Two forms of T were specified, including a GDP-bound form (T_D_) that exchanges irreversibly with GTP to generate a GTP-bound form (T_T_). An implicit nucleation site reversibly transforms non-growing T_T_ monomers into a growing form (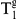) to which 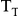 units can add.

Modeling the addition of each subunit would be impractical, as the real number of subunits per µm, N_s_ = 1625 [25], and microtubules in newborn cells can be as long as 3.5 µm. We reduced this complexity by discretizing microtubules into *N* increments each Δ*d* = 0.07 µm long. One polymerized MT subunit corresponded to *N_s_*⋅Δ*d* number of monomers, with the reaction designated as
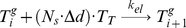
(4)In the rate expression for this reaction, the concentration dependence of T_T_ was not raised to the power 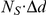 to avoid numerical instability. This simplification is reasonable because polymers with different lengths have the same velocity of elongation [15].

Growing polymers 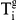 convert into shrinking ones (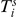) through an irreversible uncatalyzed reaction. The concentration of a microtubule of length *i* is the sum of growing and shrinking forms,
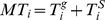
(5)The rate of this conversion reaction depends on the length of the microtubule relative to cell length, with faster rates occurring with longer polymers [26]. This length-dependence was included in rate constant *k_cat_(i)* (Table 1). The reaction for the depolymerization of shrinking polymers was treated analogously. Given the assumptions and reactions described above, the microtubule subsystem is represented by the set of ODEs
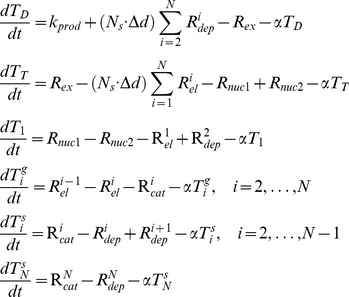
(6)subject to non-negative initial data. In the *dT_D_/dt* and *dT_T_/dt* equations, the factor 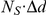 weights the depolymerization and elongation reaction rates according to the number of T_D_ subunits released or T_T_ subunits consumed per reaction event, respectively. The α-dependent terms in (6) represent dilution due to cell growth.

The microtubule and Pom1:Cdr2 subsystems interact through reactions involving Pom1 and microtubules. In fission yeast, the spatial distribution of Pom1 depends on the cellular movement of Tea1 [6], [11], [12]. In our model, Tea1 was not modeled explicitly; rather, it was lumped with Pom1. Tea1 is transported to the cell tips by “riding” on the microtubules' “plus” ends [27]. It also “walks” along microtubules, as cargo of the motor protein Tea2 [11], [12], [28]. Both processes impose a directional velocity to cytosolic Tea1. In our model, the physical transport of Tea1 is described by a Pom1 convection velocity which depends on microtubule concentrations, as calculated from the MT subsystem. This term serves to transport Pom1 to the cell tips to create the spatial gradient. Without this term, there would be no spatial gradients.

The convection velocity was composed of two terms, 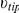 and 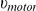, corresponding to riding and walking transport modes, respectively. These terms have the form
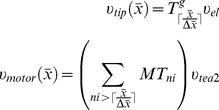
(7)In equation (7), 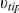 depends on the number of growing microtubules 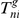 at a specific spatial point *x*, while 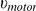 depends on the total number of microtubules passing through that point. Both are calculated from the microtubule concentrations given by system (6). 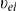 and 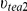 represent experimentally estimated [29] average velocities of elongating microtubules and of Tea2 moving along a MT polymer, respectively. The symbol 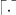 represents the ceiling function and it is used to calculate the number of normalized discrete MT subunits *ni* corresponding to a continuous length of *x µm*. Subunit normalization is described below.

### Kinetic Parameters

From the average velocity of polymer elongation 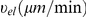 and number of tubulin subunits per micrometer (*N_S_*), it is possible to calculate the average number of tubulin subunits added to a given growing polymer per unit of time. An average polymer elongates at the rate
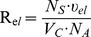
(8)where *N_A_* is Avogadro's number and *V_c_* is cell volume. Because the velocity of polymer elongation was estimated from different polymers at different cell volumes, we used an average fission yeast volume (74 µm^3^ assuming a cell radius of 1.5 µm) to calculate the reaction rate. This rate was equated to the rate-law expression for the elongation of a given polymer 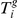 in our model. The rate was also normalized by the number of tubulin monomers included in one polymerized MT subunit. Using known values for tubulin allowed the rate-constant *k_el_* to be calculated as
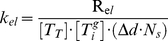
(9)The depolymerization rate constant was calculated analogously, using the average velocity of shrinkage (υ*_dp_*). In this case, the rate of depolymerization (*R_dep_*) was equated to (9) which allowed *k_dep_* to be calculated as
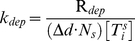
(10)


**Figure 3:**
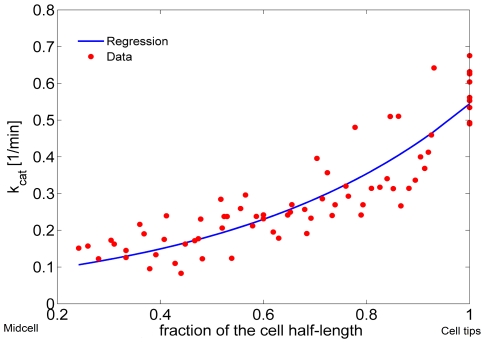
Catastrophe reaction rate. The solid line is an exponential regression (defined in Table 1) fit to the frequency of microtubule catastrophes as a function of cellular position [15].

Tischer *et al.* determined the frequency of catastrophe for MTs of different lengths by analyzing GFP-tubulin dynamics obtained from fluorescence experiments in fission yeast [15], [30]. They measured the number of catastrophe events and the MT growth time within defined cellular regions of a statistically significant number of cells of different lengths. We used these data to calculate the catastrophe rate-constant 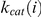 associated with the reaction used in the MT subsystem (Figure 3). An empirical exponential function (solid line in Figure 3) was fitted to the number of microtubule catastrophes per unit of normalized cell length per unit time. Because catastrophe reactions are first-order (Table 1), the catastrophe frequency estimated from the exponential regression was defined to be 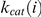.

Nucleation rate-constants *k_nuc1_* and *k_nuc2_* were set such that simulations yielded an average of 3.6 “full length” (touching the cell tips) microtubules per cell, based on the observed number of MTs in 73 cells (see supplementary material of [31]). The rate constant for GTP exchange, *k_ex_*, was set such that an apparent steady-state was reached within 10 min, as reported [32].

### Translation of the MT Subsystem into the Spatial Framework

**Figure 4:**
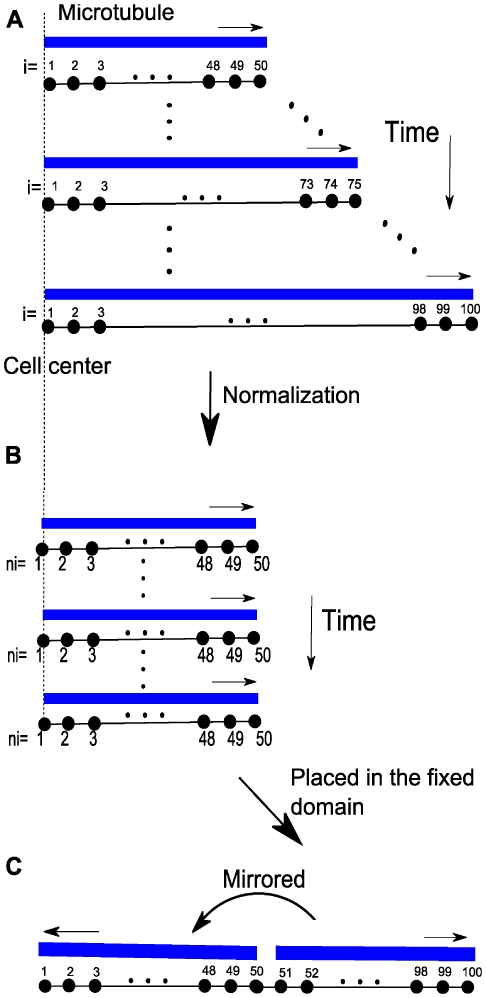
Microtubules in the growing domain framework. (A) Time dependent lengthening of microtubules within a growing cell. (B) Same as in A, after normalization. (C) Same as B, after placement in the fixed domain and mirrored.

The microtubule subsystem was created to provide “tracks” for Pom1 transport within the context of a 1D discretization of the growing cell [33]. The mathematical MT subsystem developed above is not spatially dependent even though it includes components that possess a spatial dimension. To use the time-dependent MT model in the growing cell framework, we assumed that all MTs are nucleated at the 2 central nodes (nodes *i = 50* and *i = 51* in Figure 4C) and that they grow in an antiparallel manner along the axis towards the mesh ends (*i = 1* and *i = 100*).

Cell growth was incorporated into the MT model in the following manner. As cellular volume increased, microtubules were allowed to increase their length until they reached the cell tips. Cell growth (allowing MTs to grow longer) was included in the model by increasing the number of microtubules subunits *i* for the longest polymer accordingly to the incremental growth of the cell length. During simulations, a new ODE associated with a newly added polymer was added each time the cell length increased by Δ*d* µm. The concentration of the new polymer was assumed to be zero at the moment it was introduced. Therefore, the time point representing the longest cells (where volume had doubled) contained ∼ twice as many equations as at the beginning of a simulation (Figure 4). MT lengths were normalized before being positioned into the spatial framework. Microtubule *MT_i_* was normalized into a fixed domain, *MT_ni_*, where the new number of polymerized subunits *ni* in this domain was given as
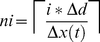
(11)In equation 11, 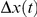 is the length of the real cellular region represented by a discretized point in the fixed domain used to solve the spatial model (Figure 2B). In this way, the results of the MT subsystem were included in the fixed mesh where the Pom1:Cdr2 subsystem was solved.

### Triggering Subsystem

In fission yeast, Cdr2 is part of a complex regulatory signaling cascade that ultimately triggers mitosis [4]. In our model, this cascade was simplified to a zeroth-order ultrasensitive switch modeled as a standard Goldbeter-Koshland function [34]. The switch is only a function of Cdr2 in the midcell region, which we presume correlates to Cdr2 in cortical nodes. The switch is indirectly affected by Pom1. Since the Pom1 concentration at the midcell region decreases with cell growth, the total Cdr2 membrane form 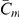 at midcell increases, triggering mitosis when the cell reaches a specific length. This presumption is supported by the co-localization of Wee1 and Cdr2 in medial cortical nodes [35] (diffusely located in the midcell region) and by the localization of Wee1 to the nuclear envelope which is also in the midcell region [4]. Wee1 inhibits mitotic entry when it is not phosphorylated, which may be controlled by cellular localization of the two proteins. Cdr2 may effectively inhibit Wee1 only when both proteins are localized to the cortical nodes.

## Results

### MT Subsystem Behavior

**Figure 5:**
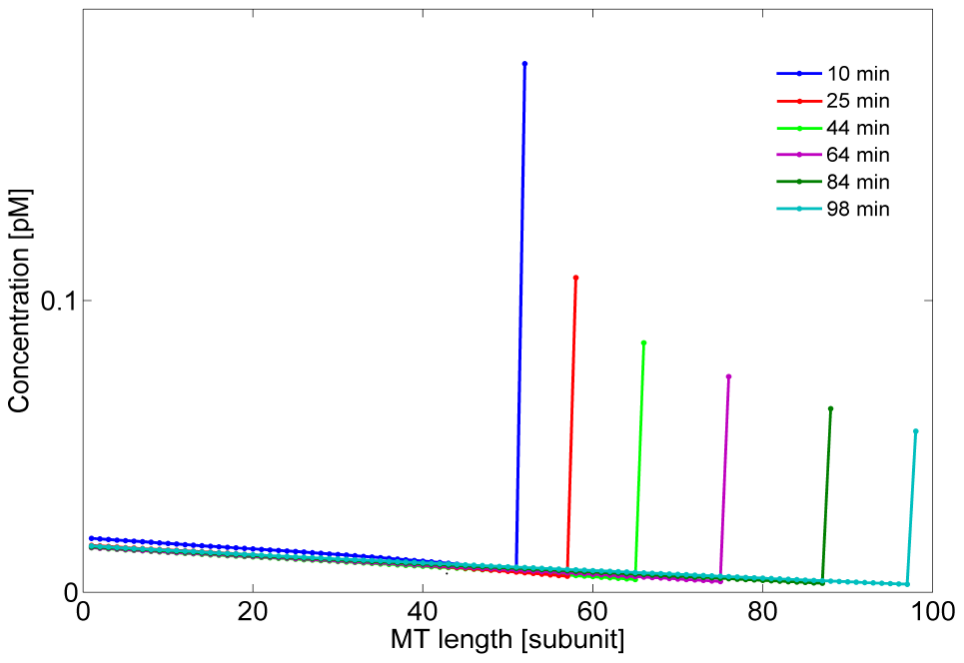
Microtubule concentrations of different lengths during cell growth. At the initial time, simulations involved 50 discretized microtubule subunits (N = 50) whereas at t = 100 min, 100 such subunits were involved. At all times, the longest microtubule in the cell had the highest concentration. The rate of subunit incorporation was not linear with time due to the exponential growth rate. The initial concentrations of T_T_, T_D_, and GTP were 7.5 µM, 0.5 µM, and 340 µM respectively.

We matched the spatial dimension of the MT subsystem to the spatial discretization interval of the Pom1:Cdr2 subsystem by making 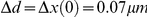. We set the maximum length of microtubules to 3.5 µm (maximum number of subunits *N* = 50) at the beginning of a simulation, representing a newborn cell. At the end of a simulation (with cell volume doubled), *N* = 100. Figure 5 shows the change in concentration of MTs as the cell grows. As the volume increased, the overall MT concentration declined exponentially with time. However, the average number of polymers touching the cell tips (3.6) was kept constant between 10 min and the end of the simulation [31]. The experimental data used to build the MT subsystem were associated only with the microtubule bundle tip - the longest MT in the bundle [15]. Thus, the associated parameters used in simulations may slightly overestimate the concentration of the longest MTs and underestimate the concentrations of shorter polymers.

### Pom1:Cdr2 Subsystem Behavior

The growing domain framework was assigned a doubling-volume time of 100 min. The cellular concentrations of Pom1 and Cdr2 were assumed to be directly proportional to the published fluorescence intensities of their spatial distributions [5]. The overall Pom1 fluorescence was normalized to 2000 copies of Pom1 at the beginning of the simulation [7]. This normalization factor was also used to estimate the number of Cdr2 copies to be 1855 in short cells. In long cells, the areas under the Pom1 and Cdr2 fluorescence curves were greater by ∼62% and 20%, respectively. These copy-number changes were used to calibrate the constant feed terms for C_c_ and P_c_ in system (2). Diffusion coefficients were estimated from literature values [7]. All other rate constants for the reaction-diffusion-convection system (2) were empirically adjusted to fit simulations to the normalized fluorescence data with greatest fidelity.

**Figure 6:**
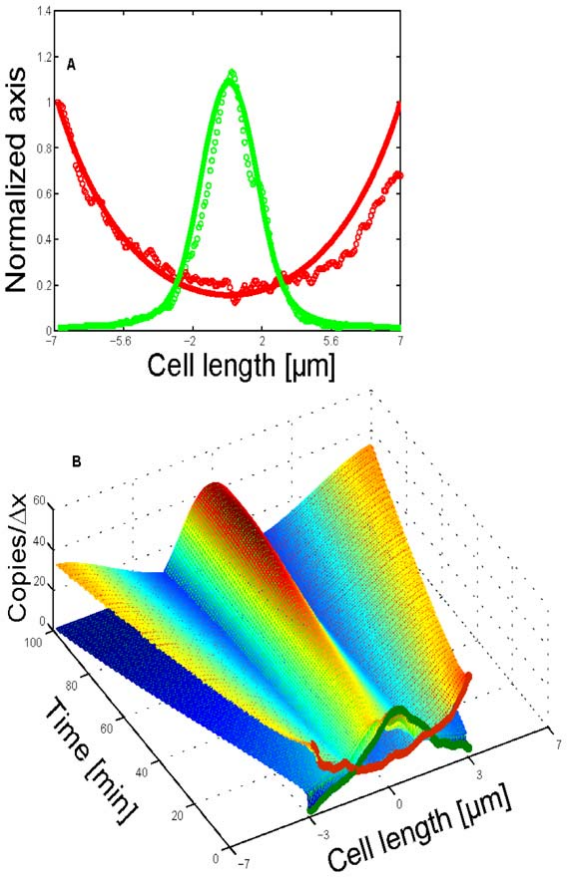
Simulation of the spatial distributions of Pom1 and Cdr2 in a growing cell during interphase. (A) The final distribution of Pom1 (red dots) and Cdr2 (green dots) in long cells ready for mitosis (data digitized from [5]). Solid lines are corresponding simulations at t = 100 min. (B) Simulations showing the time-dependent spatial profiles of total Pom1 (red) and Cdr2 (green) concentrations (both membrane and cytosolic forms). Data for the initial Pom1 and Cdr2 distributions used (for short cells at t = 0) were taken from fluorescence experiments. Simulations for this wild-type (WT) condition used the parameter values listed in Table 1 and the initial Pom1 and Cdr2 concentrations given in *Supporting Information (Text S1)*. Other parameters include 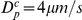, 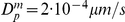, 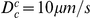; 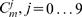, 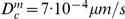, α = ln(2)/100 min^−1^, initial cell length, 7 µm, cell radius, 1.5 µm.

Numerical simulations began with a short cell (7 µm); initial Pom1 concentrations declined from the poles toward midcell while Cdr2 concentrations were maximal at midcell (Figure 6B, t = 0). Final concentrations in long cells (at t = 100 min) reproduced the fluorescence data with reasonable fidelity (Figure 6A). The convection term reflecting Pom1 transport, as defined by the microtubule dynamics, was more influential than diffusion, affording higher Pom1 concentrations at the cell tips. As the cell lengthened, the limited amount of Pom1 in the cell was predominantly delivered to the tips, leaving a deficiency of Pom1 at midcell. This allowed Cdr2 to accumulate at the cell cortex.

**Figure 7:**
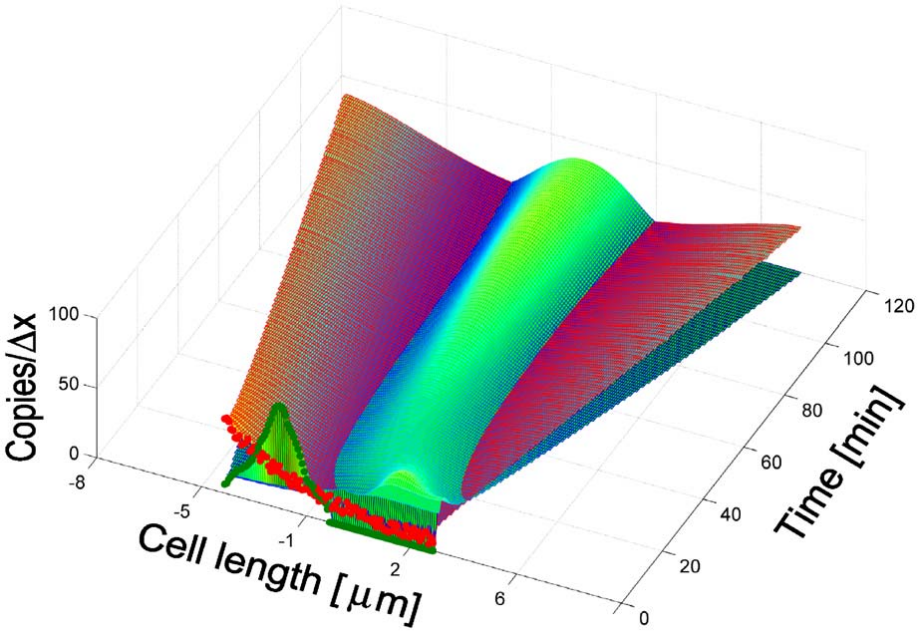
Simulation assuming an initial newborn cell distribution of Pom1 and Cdr2. Cdr2 and Pom1 were initially distributed towards one side of a short newborn cell (green and red circles at t = 0), mimicking the distribution immediately after cell division. In this distribution, the Pom1 concentration was significantly higher at the new end (cell tip created after division) than the old end. Because of this, Cdr2 rapidly shifted to the opposite cell extreme (old cell end). Gradually, the Pom1 concentration at both ends equalized, confining Cdr2 to the midcell region. Parameters were the same as in Figure 6 except for the initial distribution of Pom1 and Cdr2; values are given in *Supporting Information (Text S1)*.

Simulations with different initial Pom1 and Cdr2 distributions afforded nearly identical final spatial profiles, demonstrating model robustness (see Figure 7 for one case). These simulations also show the ability of the system to focus Cdr2 to the midcell region as part of the positioning mechanism of the eventual actomyosin ring [36], [37]. The model was sensitive to parameters related to Pom1 behavior such that formation of the Pom1 gradient dictated the general model behavior. However, different combinations of Pom1 diffusion, Pom1 transport velocities and rate constant for Pom1 detachment from the membrane produced similar overall dynamics. Thus, it is unlikely that the set of parameters used here are unique in their ability to elicit the desired dynamical behavior.

### *In Silico* Experiments

**Figure 8:**
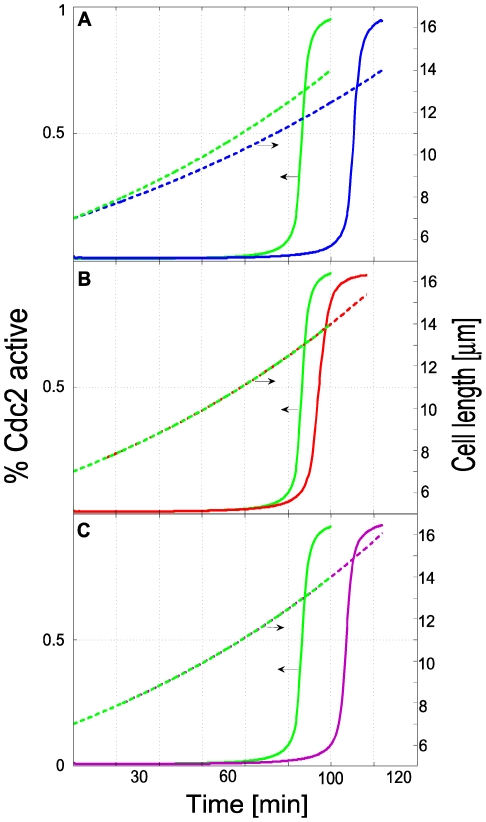
Checkpoint behavior. Cdc2 activation (solid sigmoidal curves) indicates entry into mitosis when a threshold length (dashed lines) is attained. Horizontal arrows designate the associated ordinate axis. Panels compare wild-type behavior (green) with three different experimental conditions, including actin disruption (A), microtubule depolymerization (B), and Pom1 overexpression (C). In A, the parameters used to generate the blue lines were identical to wild-type (WT) conditions (Table 1 and Figure 6) except that α was ln(2)/120 min^−1^. The red lines of B were generated using WT parameters except that the rate constant for microtubule catastrophe was increased 5-fold. The purple lines in C were generated using WT parameters except that the initial Pom1 concentration was 2-fold higher.

Simulations reflecting different experimental conditions were performed to assess the degree to which the model reproduced the cell-size checkpoint behavior of fission yeast. Reducing the growth rate α such that the time of volume-doubling was slowed from 100 to 120 min mimicked the effect of *latrunculin-A* on a wild-type cell culture. This drug disrupts actin patches and delays entry into mitosis because it increases the time required to reach the size threshold [38]. As required for size checkpoint behavior, *mitotic entry in our simulations was delayed at the reduced growth rate but it was triggered at exactly the same volume* (Figure 8A).

Next we examined the effect of reducing the microtubule concentration; this mimics the effect of methyl benzimidazol-2-yl carbamate (MBC), a microtubule-depolymerizing drug that delays the entry of WT cells into mitosis [39]. Our simulations showed a similar delay (Figure 8B). They suggest that the G2 arrest caused by microtubule depolymerization is, at least in part, a consequence of Pom1 mislocalization rather than sensing microtubule damage, as has been proposed [39]. Cells lacking microtubule interphase bundles are unable to transport Tea1 to the cell tips and consequently fail to retain Pom1 to this region. As a result, the higher Pom1 level at midcell inhibits Cdr2 more effectively, which prevents Wee1 phosphorylation and the cell remains in G2. Unlike experiments where most of the microtubules were disrupted [35], our simulations used a *reduced* MT concentration, which should have a similar effect. In our model, total disruption of microtubules would result in permanent Cdc2 inactivation (in contrast to the observed *delay* in Cdc2 activation). This difference in behavior probably arises because fission yeast contain other regulators of Cdc2 activation [18].

Finally, we increased the Pom1 concentration in simulations 2-fold, representing Pom1 overexpression mutants which exhibit a dose-dependent cell cycle delay (3-4). Mitotic entry was again delayed (Figure 8C). Further increases in the Pom1 concentration delayed the triggering time further.

## Discussion

Entering mitosis commits a cell to complete the division process. The attainment of various cell characteristics, including size, is “checked” to ensure that the cell can complete the process once started. The physico-chemical mechanisms driving such “checkpoint” behavior have remained an enigma for decades [40], [41]. However, recent experiments have suggested a possible cell-size checkpoint mechanism in fission yeast involving microtubule dynamics and the spatial gradients associated with Pom1 and Cdr2. In this study, we have developed a mathematical model of this checkpoint mechanism and have demonstrated its feasibility. To the best of our knowledge, it is the first mathematical model of a biochemically-based cell-size checkpoint mechanism in a living system. The model mimics checkpoint characteristics, replicates the spatial gradients of Pom1 and Cdr2 in growing cells, and simulates essential aspects of microtubule dynamics. It also predicts the effects of three distinct experimental conditions, including a decline of growth rate, microtubule depolymerization and Pom1 overexpression. The microtubule depolymerization simulations also suggest a possible explanation for the G2 arrest observed when cells are treated with MBC – namely that the Pom1 spatial gradient is diminished.

### Multisite Cdr2 Phosphorylation

**Figure 9:**
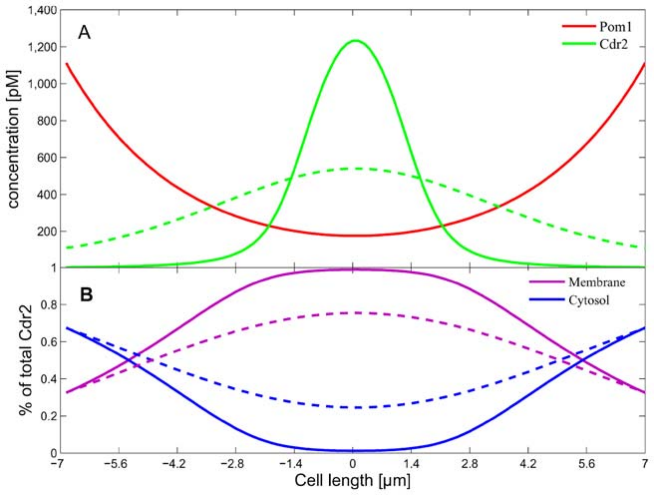
Requirement for multi-site phosphorylation. (A) Spatial concentrations of Pom1 and Cdr2 in long cells assuming Cdr2 multiphosphorylations (green solid line) and a first-order expulsion of Cdr2 from the membrane (green dashed line). The Pom1 distribution was essentially the same in both simulations. (B) Spatial distributions of Cdr2 in cytosol (blue) and membrane-bound (purple) forms correspond to the multiphosphorylation mechanism (solid lines) and the first-order expulsion mechanism (dashed lines). All other conditions used were as in Table 1 and Figure 6.

An earlier version of the model did not include the P_m_-catalyzed multiphosphylation of C_m_; rather, P_m_ was modeled to catalyze the expulsion of unphosphorylated C_m_ from the membrane in a single step. This mechanism was unable to restrict Cdr2 to the midcell region such that the Cdr2 peak was broadened relative to the data (Figure 9A, dashed green line). Including the multisite phosphorylation reactions sharpened this peak (solid green line), with 10 such reactions required to match the data. Each additional assumed phosphorylation reaction sharpened the peak incrementally. We suspect that there might be other factors controlling the Cdr2 spatial linewidth in yeast cells, and do not regard the absolute number of phosphorylation sites required here as being quantitatively accurate.

This chain of reactions sets a threshold ratio of kinase/phosphatase below which the fully phosphorylated form of Cdr2 is almost absent. In our case, it sets the ratio of 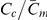 as a function of the spatial P_m_ concentration. Reactions rates were set such that the C_m_ forms dominated at P_m_ concentrations observed at midcell (Figure 9B, solid purple line). As the Pom1 concentration increased towards the cell tips, C_c_ became the dominant form in these regions. Cdr2 mostly resides on the membrane at the middle cell region because it is constantly ejected from the membrane at the tip regions by Pom1. Although a single-step reaction can achieve similar Cdr2 ratios at the cell tips, it cannot afford a sharp Cdr2 midcell peak. When different parameter values were used with the single-step reaction to afford midcell ratios similar to those of the multisite mechanism, Cdr2 was not expelled efficiently from the membrane at the cell tips, again yielding a broad Cdr2 peak.

It is clear that Pom1 phosphorylates Cdr2 *in vitro* [4], [5] but the number of phosphorylation events involved is uncertain. Ten phosphorylation events were required to sufficiently sharpen the Cdr2 peak at midcell, but other processes may contribute to the sharpness of the Cdr2 gradient in real cells such that the actual number of phosphorylation reactions may be fewer than this. An additional unidentified Cdr2 inhibitor may be involved in Cdr2 localization, the effect of which is observed in the phenotype of Pom1 mutants [4], [5]. Sterol membrane domains may also be involved in Cdr2 cellular localization [35].

**Figure 10:**
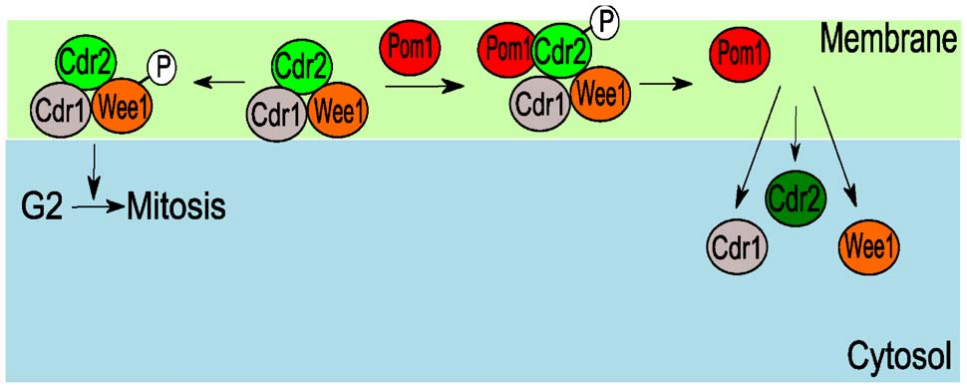
Model of cortical node localization of Cdr2-catalyzed Wee1 phosphorylation. In the absence of Pom1, Cdr2 recruits Cdr1 and Wee1 to cortical nodes where it inactivates Wee1, promoting the G2/M transition. In the presence of Pom1, cortical nodes are disrupted and Cdr2 and other cortical node components are expelled into the cytosol. The model combines the effects of Cdr2 kinase activity (recruiting Cdr1 and Wee1 to the cortical nodes) and Cdr2 localization to the cortical nodes to effectively phosphorylate Wee1. The same Pom1 mechanism that restricts Cdr2 to the midcell region could also controls the ability of Cdr2 to phosphorylate Wee1.

In our model, Cdr2 is active as a kinase only when membrane-bound, which we interpret as being when it resides in cortical nodes. Our model also assumes that Pom1 inhibits Cdr2, not by inhibiting its kinase activity, but by detaching it from these nodes (Figure 10). This mode of activation/deactivation has some experimental support. First, Cdr2 is essential in forming cortical nodes [4]. Wee1 and Cdr1 (a direct inhibitor of Wee1) localize to these nodes only in the presence of Cdr2 [4]. Cdr1 might only efficiently phosphorylate Wee1 once both proteins are in the nodes, as their local concentrations would be far greater than when they are in the cytosol [42]. Rate enhancement could be as high as the ratio of the cytosol volume to the cortical node volume [42], assuming first-order dependences. Importantly, Pom1 phosphorylates the non-catalytic terminus of Cdr2 which is responsible for attaching Cdr2 to the membrane [4], [5], [35]; this suggests that Pom1 is not affecting the kinase activity of Cdr2. Also, cortical nodes are disrupted and entry into mitosis is delayed when a mutant Pom1 binds to the cortex at the midcell region [4], [5]. Cells with defective Cdr2 membrane localization but intact kinase activity respond differently to nutrient starvation relative to wild-type cells [35], suggesting that Cdr2 localization is closely related to its function.

Cdr2 midcell fluorescence increases modestly by the end of G2 phase [5], but this has not been considered previously as being associated with the mechanism that links cell size to cell cycle events. If there was no increase of the membrane-bound form of Cdr2 at midcell, the Cdc2 triggering mechanism used here could not function appropriately. Whether the mitotic trigger would be sufficiently robust with only the modest observed increase of Cdr2 fluorescence at the midcell region [5] is uncertain. If a switch-like mechanism is responsive to such small concentration changes, one would expect a large variability of cell lengths due to genetic noise [43], which is not observed. However, other mechanisms may enhance stability and buffer the system against such noise. Positive and double-negative feedback loops can add bistability robustness to the cell size checkpoint [44], and there are other unidentified components involved in cell-size sensing [4], [5], [45]. In any event, our reaction-diffusion-convection system provides a reliable framework for Pom1 and Cdr2 spatial localization where different hypotheses for the link between cell size and cell cycle can be explored.

Finally, we have considered whether the Pom1-dependent cell size checkpoint mechanism could be more generally used in eukaryotic cells. Pom1 is a member of the DYRK (dual-specificity tyrosine-regulated kinase) family. These proteins are involved in cell cycle regulation and control of cell proliferation and differentiation [46]. Although it is unclear that other Pom1 homologs are used in cell-size checkpoint mechanisms, this possibility is intriguing. Such mechanisms would likely involve size-dependent shifts in protein spatial gradients. Cell size and shape are reportedly involved in controlling the phosphorylation states of cellular components [47] which may be involved in size sensing. Efforts should be made to identify new size-related proteins that are connected to the cell cycle machinery and that exhibit spatial concentration gradients. Such proteins may play key roles in cell-size checkpoint mechanisms.

## Supporting Information

Text S1 Initial Pom1 and Cdr2 data.
(0.01 MB TXT)
